# Stepwise transvenous lead extraction due to pacemaker pocket infection following lactational mastitis complicated with breast abscess

**DOI:** 10.1186/s13006-024-00633-0

**Published:** 2024-04-19

**Authors:** Lidija Poposka, Dejan Risteski, Dimitar Cvetkovski, Bekim Pocesta, Filip Janusevski, Zhan Zimbakov, Ivan Trajkov, Dime Stefanovski, Mateja Logar, Jus Ksela

**Affiliations:** 1 University Clinic for Cardiology, Skopje, North Macedonia; 2 Faculty of Medicine, Ss. Cyril and Methodius University in Skopje, Skopje, North Macedonia; 3 Clinical hospital “Acibadem Sistina” Skopje, Skopje, North Macedonia; 4 Department of Cardiovascular Surgery, University Medical Centre Ljubljana, Ljubljana, Slovenia; 5 Clinic of Infectious Diseases and Febrile Illnesses, University Medical Center Ljubljana, Ljubljana, Slovenia; 6 Faculty of Medicine, University of Ljubljana, Ljubljana, Slovenia

**Keywords:** Lactational mastitis, Breast abscess, Pacemaker pocket infection, Transvenous lead extraction

## Abstract

**Background:**

Lactational mastitis is a common painful and debilitating inflammation of breast tissue, generally treated conservatively or with pus puncture in case of breast abscess. However, treating mastitis in patients with implantable surgical material located in the affected breast region can be extremely challenging. We present an unusual case of lactational mastitis complicated by pacemaker pocket infection in a breastfeeding mother.

**Case presentation:**

A 35-year-old pacemaker-dependent female developed lactational mastitis seven weeks postpartum. Initially, the condition was treated conservatively with analgesics and antibiotics. After abscess formation, pus was aspirated using fine-needle aspiration technique. Four weeks after mastitis resolution, pacemaker pocket infection developed. According to current cardiovascular implantable electronic device infection treatment guidelines a complete surgical extraction of the entire electronic system, followed by targeted antibiotic treatment and reimplantation of a new device after infection resolution, was recommended. However, after thorough discussion with the young woman and her family and after detailed review of surgery-related risks, she declined a potentially high-risk surgical procedure. Thus, only the pulse generator was explanted; pacing leads positioned in the sub-pectoral pocket; new pacemaker implanted on the contralateral side and broad-spectrum antibiotic therapy continued for six weeks. After breastfeeding cessation, and with chronic fistula development at the primary pacemaker implantation site, the possibility of delayed surgical intervention including complete extraction of retained pacemaker leads was again thoroughly discussed with her. After thoughtful consideration the woman consented to the proposed treatment strategy. A surgical procedure including transvenous lead extraction through the primary implantation venous entry site, using hand-powered bidirectional rotational sheaths, was successfully performed, removing all retained leads through the left subclavian venous entry site, and leaving the fully functional and clinically uninfected pacemaker on the contralateral site intact.

**Conclusion:**

Although patients’ decisions for delayed extraction in a case of cardiovascular implantable electronic device infection should be discouraged by attending physicians and members of interdisciplinary teams, our case shows that a stepwise treatment strategy may be successful as a bailout clinical scenario in patients with specific requests, demands and / or clinical needs.

## Background

Lactational mastitis is defined as cellulitis of the interlobular connective tissue within the mammary gland and is characterized by physical, chemical, and bacteriological changes in the milk [1]. This painful and debilitating multi-etiological inflammatory condition ranges in clinical spectrum from focal inflammation with minimal systemic symptoms, such as local erythema, tenderness, engorgement, and severe breast pain accompanied by flu-like symptoms to abscess formation, bacteriemia, and sepsis [1, 2].

Although the majority of affected women are treated conservatively and pharmacologically or potentially with pus puncture or surgical drainage in case of abscess formation [2, 3], treatment of lactational mastitis and mastitis related complications in patients with surgical implantable material in the breast or close to the breast region — such as silicone breast implants or cardiovascular implantable electronic devices (CIED) — can be extremely challenging, necessitating individualized and patient-specific treatment strategies [2–6].

In this report, we present a rare case of secondary pacemaker pocket infection following lactational mastitis in a young, pacemaker dependent, breastfeeding female.

## Case report

A 35-year-old Caucasian female had her first pacemaker implanted in 2005 at the age of 18 years due to a complete atrioventricular block. During the primary implantation, a pocket was created in the left sub-clavicular region, and the pulse generator placed under the subcutaneous space above the fascia of the left pectoral muscle (i.e., supra-fascially). In 2006, she had a new ventricular lead implanted due to a primary ventricular lead dysfunction. During the new lead implantation, the primary lead was left in the device pocket. In 2012, she underwent an uneventful pacemaker generator replacement due to battery depletion. In the following years, she was managed on an outpatient basis with an uneventful clinical course, with proper pacemaker function, and no reported pacemaker related issues.

In 2021 she gave birth to a healthy child. This was her first pregnancy. She conceived spontaneously and received routine prenatal care with no documented complications during pregnancy. Labour started spontaneously at term (39 weeks of gestation) with regular uterine contractions. The foetus was in cephalic presentation. She received no antibiotic prophylaxis. Labour progressed normally and resulted in a vaginal birth of an appropriate-for-gestational-age birth weight neonate with an Apgar score of nine at birth and ten at five minutes of life. She was hospitalised for three days after birth. The postpartum period until discharge from the maternity hospital was uneventful, with early initiation of breastfeeding without complications. However, seven weeks postpartum she developed lactational mastitis of the lower outer quadrant of the left breast with local erythema, tenderness, engorgement, and local pain. A conservative and pharmacological treatment strategy with continuation of breastfeeding, cooling of the affected area, and analgesic therapy was advised. However, with the inflamed upper outer breast quadrant and left sub-clavicular region, and with the woman becoming febrile, antibiotic treatment was advised. Since the young breastfeeding mother was reluctant to take empirically selected antistaphylococcal penicillin, amoxicillin / clavulanic acid was prescribed for seven days, according to local guidelines.

Despite antibiotic treatment, an abscess evolved in the upper outer breast quadrant, as determined by clinical examination (showing signs of local edema and erythema, warmth and severe pain) and soft-tissue ultrasound (appearing as an ill-defined mass with central hypoechogenic area and eccentrically thickened walls and displaying internal septation and debris). The abscess was punctured using fine-needle aspiration technique and purulent material sent for microbiological analysis. *Staphylococcus aureus*, sensitive to methicillin as well as to high doses of amoxicillin / clavulanic acid, was detected. With the isolate available, it was possible to persuade the patient to switch to the most optimal antimicrobial therapy (flucloxacillin for an additional 14 days), after which the signs and symptoms of mastitis gradually resolved. However, four weeks later, a clinically evident pacemaker pocket infection with local erythema, pain, and palpable fluctuation around pulse generator developed. The woman was advised by an interdisciplinary medical team including attending cardiologist, consultant infection disease specialist and cardiovascular surgeon according to current CIED infection treatment guidelines, to undergo a complete pacemaker system extraction strategy, using a well-established transvenous lead extraction (TLE) technique. However, after comprehensive discussion including the young breastfeeding mother, her family and the multidisciplinary team, she did not consent to a potentially risky TLE procedure. Thus, only the pacemaker pulse generator was explanted; three retained pacing leads (two ventricular and one atrial) were positioned in the left sub-pectoral pocket; a new dual chamber pacemaker implanted on the contralateral side and a broad-spectrum antibiotic therapy with cefepime continued for six weeks.

**Fig. 1:**
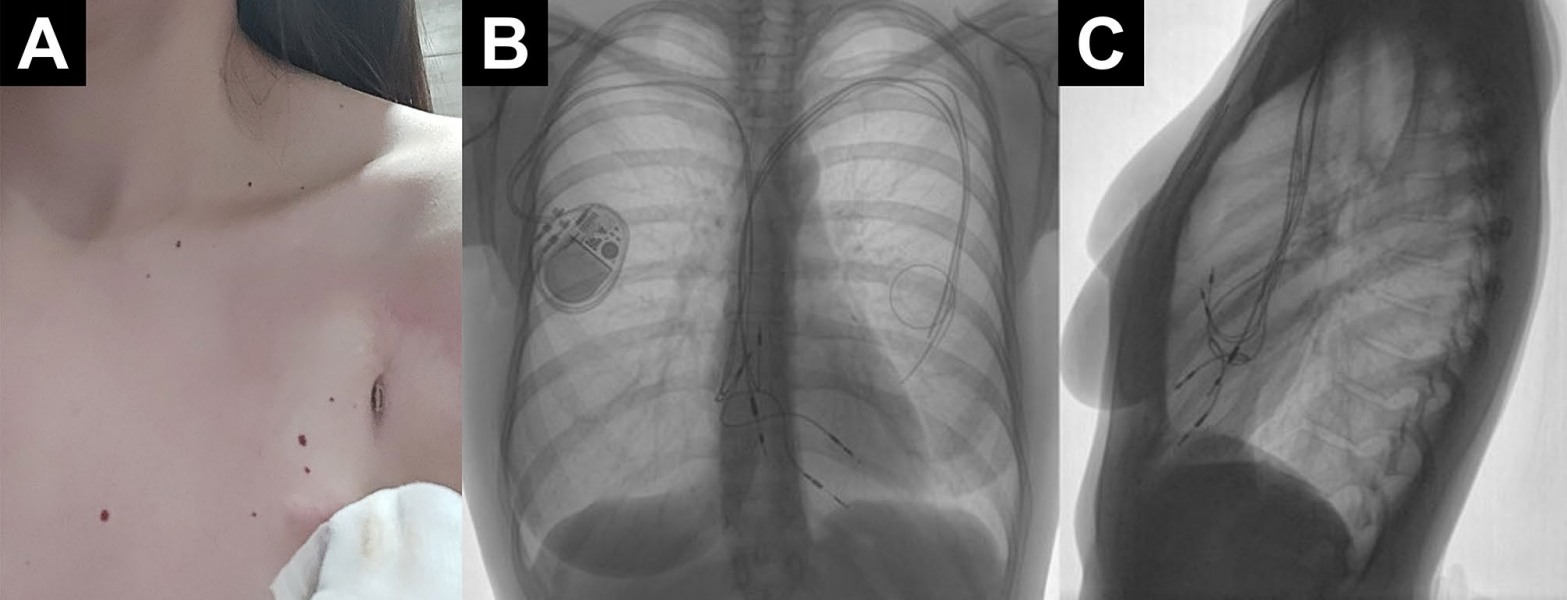
Photograph and chest x-ray of case at 16 months postpartum. Photograph of individual in case study at 16 months postpartum showing a chronic skin fistula above the left-sided pocket (**A**), and antero-posterior (**B**) and lateral (**C**) view of chest x-ray made before the retained leads extraction. Retained left-sided leads and completely functional right-sided pacemaker system are clearly visible on the chest x-ray

In the following months, a chronic skin fistula above the left-sided pocket developed (Fig. 1).

Due to the high possibility of a new infection of a fully functional right-sided pacemaker system as a result of chronic left-sided leads infection, the delayed surgical treatment strategy involving TLE was again strongly advised by the same multidisciplinary team. In the light of new clinical circumstances, such as cessation of breastfeeding four months prior and chronic left-sided leads infection potentially leading to potentially fatal right-sided pacemaker system complications, another thorough discussion was performed with the woman and her family. After a detailed review of surgery-related risk, she did consent to the proposed surgical strategy. Therefore, we opted for a now more complex TLE procedure of the three retained left-sided leads while leaving the fully functional and clinically uninfected right-sided pacemaker system intact.

**Fig. 2:**
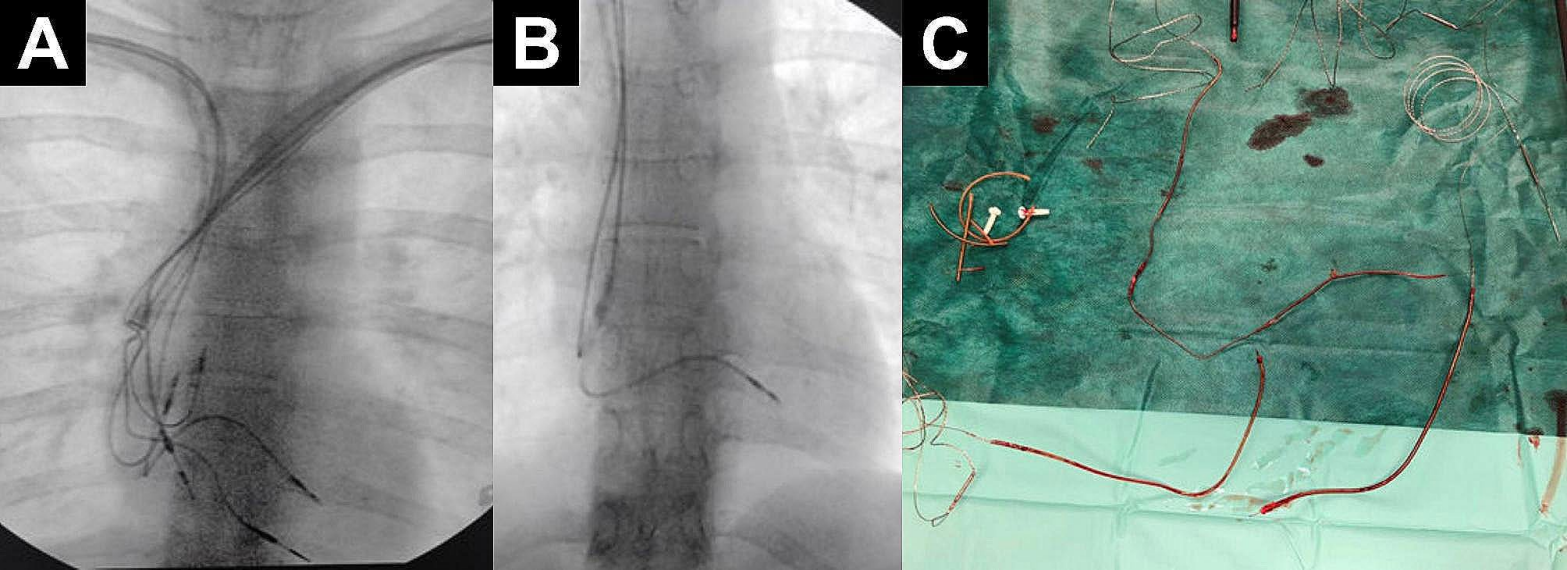
Intraprocedural fluoroscopic images showing a successful transvenous lead extraction procedure. Intraprocedural fluoroscopic image showing a hand-powered dilating sheath freeing the lead from adhesions (**A**), completely intact right-sided pacemaker system after successful transvenous lead extraction procedure (**B**) and three completely extracted leads, attached to the locking stylets (**C**)

The surgical procedure was performed at the University Clinic for Cardiology, Skopje, Northern Macedonia in 2022, under general anaesthesia with invasive patient monitoring, continuous transesophageal echocardiogram surveillance and with utilization of high-quality fluoroscopy as described elsewhere [7]. Next, an incision was made over the scar from previous surgeries, and the chronic fistula was excised. Subsequently, the abandoned leads’ ends were dissected and a standardized TLE procedure was performed using Liberator® Beacon® Tip locking stylet (Cook Medical), One-Tie® Compression Coil (Cook Medical), and Evolution Shortie RL® and Evolution RL® (Cook Medical) hand-powered bidirectional dilator sheaths (Fig. 2) through primary implantation venous entry site (i.e., left subclavian vein), as described in detail elsewhere [7].

Of note, during the procedure, particular care was paid to fully functional leads from the right-sided pacemaker system to preserve them and to avoid any need for additional high-risk surgical procedures. After the surgery, the patient was monitored in the intensive care unit and bedside echocardiography was performed to exclude damage to right-sided heart structures and because of the presence of a pericardial effusion. The young woman was discharged the next day and broad-spectrum antibiotic therapy was continued for 14 days.

The tips of the extracted electrodes along with the tissue attached to the extracted electrodes (Fig. 2) were sent for microbiological and histopathological analysis. Histopathological features were typical of sterile vegetation with no microorganisms isolated.

Twelve months after the procedure, the young female patient is clinically stable with no signs of local or systemic infection; with a completely healed left-side skin incision with no signs of residual chronic fistula; a fully functional right-sided pacemaker system; and is managed on an outpatient basis.

## Discussion

Lactational mastitis is a painful and debilitating inflammation of breast tissue that has an important impact upon breastfeeding duration and frequency in many lactating mothers [1]. Affected individuals usually develop a swollen, tender, and painful breast with a focal area of erythema, and flu-like symptoms including fever and chills [2]. Yet, if the condition is managed poorly or left untreated, a breast abscess can form along with bacteremia and sepsis [1–3].

Whereas mastitis is a recognized cause of fever during the postpartum period, its precise incidence and epidemiology in the breastfeeding population is not well defined and can vary tremendously, depending on numerous factors such as geographic location, cultural practice, and breastfeeding preferences [1]. It is estimated that around 10% to up to 33% of breastfeeding women will experience mastitis at some point during their lactation period [2, 3]. The incidence of lactational mastitis tends to be highest within the first 6 to 12 weeks postpartum and gradually decreases over time [1–3]. The high incidence in the initial period is often related to women’s early adjustments to breastfeeding, as well as issues related to skin injuries, and ineffective milk removal [2, 3]. Furthermore, certain determinants have been identified as risk factors for lactational mastitis, including previous history of mastitis, poor breastfeeding techniques, cracked and sore nipples, incomplete breast emptying, infrequent or missed breastfeeding sessions, lower maternal immune status, and fatigue secondary to stress and sleep deprivation [1, 2]. Although the exact etiology in each patient is difficult to determine, hyperlactation causing milk duct narrowing and consequent breast engorgement or intrusion of baby’s mouth bacteria through sore and cracked nipples into the milk ducts are believed to be the most common causes of the disease [2]. A blend of different pathogens is involved in the bacterial form of lactational mastitis, with *S. aureus* being the most common pathogen [3].

In the majority of patients with lactational mastitis, management of the disease is rather straightforward, including continuation of breastfeeding, cooling of the affected breast area, reverse pressure softening, and usage of nonsteroidal anti-inflammatory drugs and antibiotics in cases of severe pain and signs of systemic inflammation. If mastitis progresses to abscess formation, pus puncture using fine-needle aspiration technique or surgical incision and pus drainage is indicated as soon as possible [2, 3]. However, treatment of lactational mastitis and mastitis related complications in patients with implantable material in the breast or close to the breast region (i.e., silicone breast implants, CIED or implantable loop recorders) can be extremely challenging [2, 3], requiring patient-specific treatment strategies. This is especially so in cases of surgical implantable material infection which necessitates complete removal from the body [2–10].

Evidence indicates that lactational mastitis is fortunately only rarely complicated with secondary infection of implantable surgical material that lies in or near the affected breast [2–10], however such a clinical course is the most plausible scenario in the breastfeeding mother described above. We strongly believe that severe lactational mastitis in this woman was complicated with secondary pacemaker pocket infection through direct bacterial contamination. Other causes of pacemaker pocket infection, such as infection through the hematogenous route during an episode of bacteremia or late clinical presentation of bacterial contamination during previous surgeries seem highly unlikely. This is mainly due to the absence of retained leads endocarditis and the long and completely uneventful postoperative course [4–6].

According to current American Heart Rhythm Society and European Heart Rhythm Association guidelines for the treatment of CIED infection (including device pocket infection), prompt extraction of the entire electronic system, followed by targeted antibiotic treatment and reimplantation of a new device after infection cessation is indicated in virtually all affected individuals mainly due to high mortality (i.e., up to 35%) following untreated CIED infection [5–12].

Over the last decade, TLE has greatly advanced and is currently regarded as the premier surgical method when CIED removal is indicated: it provides a complete, safe and highly effective mode of implanted leads extraction with reported success rates ranging from 83.3 to 97.6% and major complication rates ranging from 1.5 to 2.4% [11–13]. Although the reported TLE complication rate is relatively low, especially in dedicated, high-volume centres, the nature of potential complications (i.e., anaesthesia-related severe adverse events, sepsis, respiratory distress, cardiac or vascular avulsion or tear requiring pericardiocentesis or open-heart surgery, and even death in 0.5% of patients [13, 14]), is non-negligible and may direct the patient’s decision-making process. Regardless of the clear recommendations from the scientific community it is sometimes difficult for some patients to decide whether to undergo a potentially risky surgical procedure of complete lead extraction or to leave infected electrodes in-situ and combine a less risky but incomplete surgical procedure with long-term antibiotic therapy, as was the case in this young breastfeeding mother [5, 6, 12].

Every TLE is regarded as a complex surgical procedure, yet, several clinical factors, such as very long dwell time (> 10 years); young age at primary implantation; preoperative anemia; and damage to remnant leads during previous surgery; have been reported as risk factors for even more demanding, technically more challenging, and potentially unsuccessful procedures [7, 13–16]. This was also the case in the young mother described above– she was only 18 years old when she underwent the first pacemaker implantation; the dwell time was 15 years (combined dwell time of all electrodes was 74 years); and all three leads were at least partially damaged during previous surgery. Of note, a fully functional pacemaker implanted from the contralateral side represented an additional risk factor for clinical failure of the TLE procedure [6]. Performing a TLE procedure by leaving the contralateral pacemaker system intact is technically even more demanding and raises the odds of an unsuccessful treatment outcome [12–16].

Since the current patient-orientated medical culture appreciates and encourages shared-decision-making and values each individual’s autonomy, especially among women in the antenatal and postpartum time-periods, our team respected the young mother’s requests and complied with her wish not to undergo a complete pacemaker system removal, despite having clinically evident pacemaker pocket infection. However, in everyday clinical practice we usually try to discourage our patients from declining such surgical strategies [6, 12]. Patients should be strongly advised and encouraged by all members of the multidisciplinary team to undergo a complete extraction to maximize the treatment success and to minimize the possible short- and long-term complications [6, 12]. By delaying consent for the indicated surgical procedure due to — according to her understanding — unacceptably high surgery-related risks, this young breastfeeding mother has unfortunately increased the likelihood of an unsuccessful treatment outcome and risks for potentially severe and life-threatening complications following a non-optimal treatment scenario. Even though our stepwise surgical therapy has resulted in an overall successful treatment outcome, such treatment strategies should be used only as a bailout clinical scenario if consent to recommended treatment is refused for any reason.

## Conclusion

We present an unusual and — to the best of our knowledge — so far, unreported clinical scenario of a young female patient with lactational mastitis complicated with pacemaker pocket infection who did not consent to complete pacemaker system removal due to the potential surgery-related risks which she was not comfortable to accept as the mother of a new-born child. Thus, after a thorough discussion with the woman and her relatives, and after a detailed review of surgery-related risks, a stepwise surgical strategy was strongly recommended to her with a TLE procedure performed more than a year after pacemaker pulse generator extraction and retained leads pending in the sub-pectoral pocket. Although a patient’s decision for such a treatment strategy should be strongly discouraged by attending physicians and members of a multidisciplinary team, our case shows that it may be successfully used as a bailout clinical scenario in selected patients.

## Data Availability

No datasets were generated or analysed during the current study.

## Abbreviations

CIED: Cardiac implantable electronic device
TLE: Transvenous lead extraction
